# Overview of Virulence and Antibiotic Resistance in *Campylobacter* spp. Livestock Isolates

**DOI:** 10.3390/antibiotics12020402

**Published:** 2023-02-17

**Authors:** Iulia Adelina Bunduruș, Igori Balta, Lavinia Ștef, Mirela Ahmadi, Ioan Peț, David McCleery, Nicolae Corcionivoschi

**Affiliations:** 1 Faculty of Bioengineering of Animal Resources, University of Life Sciences King Mihai I from Timisoara, 300645 Timisoara, Romania; 2 Bacteriology Branch, Veterinary Sciences Division, Agri-Food and Biosciences Institute, Belfast BT4 3SD, UK

**Keywords:** *Campylobacter* virulence, antibiotic resistance, virulence, foodborne pathogens, infection, gastroenteritis

## Abstract

*Campylobacter* remains the most prevalent foodborne pathogen bacterium responsible for causing gastroenteritis worldwide. Specifically, this pathogen colonises a ubiquitous range of environments, from poultry, companion pets and livestock animals to humans. The bacterium is uniquely adaptable to various niches, leading to complicated gastroenteritis and, in some cases, difficult to treat due to elevated resistance to certain antibiotics. This increased resistance is currently detected via genomic, clinical or epidemiological studies, with the results highlighting worrying multi-drug resistant (MDR) profiles in many food and clinical isolates. The *Campylobacter* genome encodes a rich inventory of virulence factors offering the bacterium the ability to influence host immune defences, survive antimicrobials, form biofilms and ultimately boost its infection-inducing potential. The virulence traits responsible for inducing clinical signs are not sufficiently defined because several populations have ample virulence genes with physiological functions that reflect their pathogenicity differences as well as a complement of antimicrobial resistance (AMR) systems. Therefore, exhaustive knowledge of the virulence factors associated with *Campylobacter* is crucial for collecting molecular insights into the infectivity processes, which could pave the way for new therapeutical targets to combat and control the infection and mitigate the spread of MDR bacteria. This review provides an overview of the spread and prevalence of genetic determinants associated with virulence and antibiotic resistance from studies performed on livestock animals. In addition, we have investigated the relevant coincidental associations between the prevalence of the genes responsible for pathogenic virulence, horizontal gene transfer (HGT) and transmissibility of highly pathogenic *Campylobacter* strains.

## 1. Introduction

Virulence and antibiotic resistance frequently arise at the same time, but the relationship between the two at the genetic level has been largely overlooked. Studies have associated virulence with multi-drug resistance in multiple animal infection models [1], but the regulation of virulence in the presence of antibiotic resistance remains unclear [2]. Antibiotic resistance and virulence can manifest through different forms of adaptative, innate and acquired resistance. The regulation of genes coding for virulence and antibiotic resistance is complex and interconnected, while environmental factors influence gene expression, making the regulation of these genes difficult. Common mechanisms involved in regulation serve as a link between antibiotic resistance and virulence [2]. Bacteria’s great ability to adapt to various environmental conditions, coupled with antibiotic pressure, has led to the rise and dispersion of antibiotic-resistant bacteria. The relationship between antibiotic resistance and virulence in bacterial pathogens depends on the species of bacteria, the specific mechanisms of virulence and resistance, the host and the ecological niche [3]. To effectively control the spread of antibiotic resistance, the control of increased virulence is also necessary, as it may evolve concurrently with antibiotic resistance. A better understanding of the interrelated regulation of antibiotic resistance and virulence can lead to more directed and specific drug treatments [2].

Hospital-acquired diseases caused by antibiotic-resistant bacteria derive from complex interactions between various dynamic factors, including fitness costs, bacterial pathogenicity and selective pressures from antibiotic therapy. The rise and aftermath of mutations that confer antibiotic resistance are shaped by these interactions. Mutations that increase antibiotic resistance can either cause decreased or increased pathogenic potential. Further research is needed to elucidate the origin of altered virulence potential in pathogens displaying antibiotic resistance and to better understand the modulating implications of resistance traits on disease outcomes. Additionally, it is important to note that the disease process itself generates selective pressures that constrain the resistance spectrum [4]. For example, in response to different antibiotic levels, *Acinetobacter baumannii* augments its virulence, altering its normally low virulence into one that induces lethal disease [5]. This ability was observed in the presence of erythromycin and chloramphenicol resistance, which temporarily enhance the production of capsular exopolysaccharide via triggering a transcriptional response by targeting the 50S ribosome [6]. Research has shown that antibiotic use can affect the expression of virulence genes. However, the relationship between genes that confer virulence and antibiotic resistance is highly intricate. Previously, it was believed that these events were regulated separately, but recent studies unveiled that genetic regulation is closely connected and intertwined [4,7,8].

**Figure 1:**
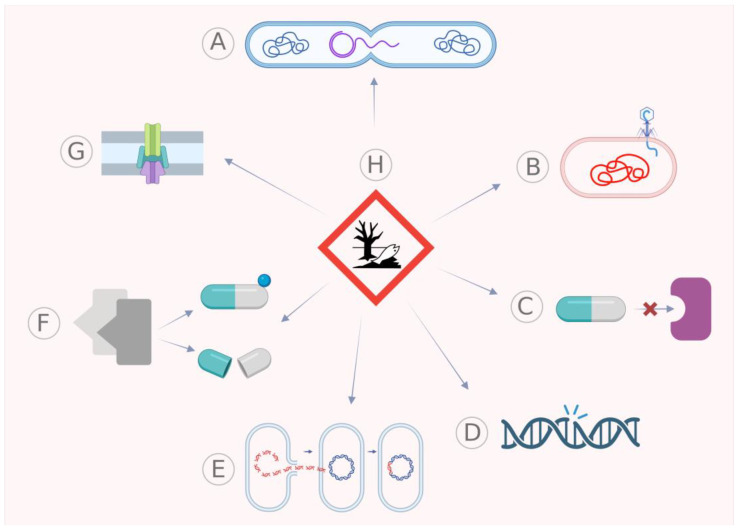
Various mechanisms and factors that play a significant role in the regulation of genes encoding virulence and antimicrobial resistance. (**A**) Conjugation; (**B**) transfection; (**C**) alteration of the antibiotic target; (**D**) elevated mutation rates; (**E**) transformation; (**F**) inactivation of antibiotics; (**G**) efflux pump; with (**H**) environmental factors exerting a secondary and ultimate effect on all the others. Created with Biorender.com.

The development of “new-generation” antibiotics, which are mostly derivatives of already existing classes, has been followed by the rise of antibiotic-resistant bacterial strains [9]. The spread of antimicrobial resistance is largely facilitated by horizontal gene transfer (HGT), with acquired resistance primarily propagated through three mechanisms: mobile genetic elements, the acquisition of DNA originating from the environment, or transfection by bacteriophages [10]. The acquisition of antibiotic resistance can not only impact virulence but also lead to the co-selection of both traits through mobile genetic elements through mechanisms of transfer, such as conjugation, transformation and transduction among bacterial strains of the same or different species. Plasmids, which are extra-chromosomal elements, can also confer attributes such as resistance, virulence and persistence to bacteria, aiding their adaptation to various environments and niches [11]. The regulation of virulence-encoding genes is intricately intertwined with that of genes responsible for antimicrobial resistance, and both are indirectly and ultimately influenced by environmental factors [2]. Figure 1 provides a comprehensive illustration of the mechanisms and factors that govern the regulation of this gene set.

Concerns are rising regarding the presence of antibiotic-resistance genes and virulence-associated genes in food-producing animals and humans. These genes, considered environmental pollutants, can cause severe complications for animals and humans. Misuse and overuse of antibiotics are accelerating the problem of increasing antimicrobial resistance and posing a threat to all inhabitants on earth [12]. Thus, the emergence and spread of ARGs have become the primary obstacle in managing infectious diseases [13]. It is crucial to identify antibiotic resistance patterns and the pathogenicity of bacteria to minimise complications and treatment failure in infections caused by multidrug-resistant pathogens. Recent research found that waterfowls may act as a significant reservoir of these strains, which can spread into the environment through water, meat, carcasses and droppings, posing a threat to human health. Therefore, surveillance programs are needed to regulate the use of antibiotics and ensure clinical prescriptions are based on antibiograms in both veterinary and human medicine [14].

It has been observed that various species within the *Campylobacter* genus can exchange genetic material with one another, as well as with other bacterial genera [15,16,17,18,19,20]. This facilitates the transfer of genes that enhance the virulence of *Campylobacter* and allow it to withstand exposure to antibiotics. This bacterial genus is widely present in multiple hosts, including humans, animals, food products and the environment. The interconnected nature of these factors presents challenges for effectively addressing the potential negative consequences for human and animal health. Exposure to the recent relevant discoveries concerning virulence factors and emphasising the genomic diversity and plasticity of *Campylobacter* makes it inconceivable to control its spread and evolution. However, monitoring the molecular changes could pave the way to a better understanding of the pathogenicity of evolution and virulence in humans, animals and the environment. Clearly, our review is not aiming to identify a genetic relationship between antibiotic resistance and virulence since it has been shown that there is no linkage between virulence and antibiotic resistance at either the chromosomal or plasmid levels [21], but rather try to provide the coincidental data most recently published. This review only provides an overview of the spread and prevalence of genes associated with virulence, including the genes conferring resistance to antibiotics in *Campylobacter* species belonging to different sources and hosts. We also showed associations between the prevalence of the genes responsible for pathogenic virulence, horizontal gene transfer (HGT) and transmissibility of highly pathogenic *Campylobacter* strains.

## 2. *Campylobacter* spp. Overview

The *Campylobacter* genus represents a well-known group of foodborne pathogens responsible for causing self-limited gastroenteritis in humans, which sometimes requires antimicrobial treatment due to the potential for serious complications [22]. In the European Union, campylobacteriosis was the most frequently reported zoonosis in humans, with a total of 127,840 confirmed cases and with the majority of *Campylobacter* spp. being detected in all major animal categories, including pigs, broilers, small ruminants, cattle, cats and dogs [23]. However, data from the last 20 years also includes livestock as a reservoir for the emergence of methicillin-resistant *Staphylococcus aureus*, *Clostridium difficile* and *Escherichia coli*, besides *Campylobacter* and *Salmonella*, with an increased impact on human health. Mitigation measures, such as increased biosecurity or vaccines, are proposed as solutions to reduce antimicrobial use in livestock [24]. Natural antimicrobials (e.g., plant extracts) could represent one of the solutions able to reduce antibiotic usage in livestock since they have been shown to reduce *Campylobacter* spp. poultry gut colonisation and virulence, both in vitro and in vivo [25]. The positive impact of these mitigation measures extends to other bacterial species, such as *Staphylococcus aureus* and *Clostridium difficile* [26] and could potentially prevent and alleviate viral infections [27]. These novel solutions are very useful to farmers as recently it has been reported that cattle and chickens are an important reservoir of campylobacters, associated with human disease, and resistant to erythromycin, ampicillin, tetracycline, nalidixic acid and ciprofloxacin [28]. Measures implemented at the slaughterhouse, rather than at the farm, must also be considered since contamination can also occur in such environments [29]; however, the management involved in farming activities seems to take priority in preventing *Campylobacter* spp. spreading in livestock [30]. The availability of such mitigation measures will prevent the overuse of antibiotics in livestock production, which lead to a high prevalence of *Campylobacter* spp. from chickens and pigs to macrolides suggesting that continuous surveillance is required to reduce the antibiotic-resistant campylobacteriosis in livestock and humans [31].

Infections with *Campylobacter* spp. can have various clinical consequences in animals as well, including reproductive failures in ruminants. For example, *C. fetus* subsp. *venerealis* induces cattle infertility, *C. fetus* subsp. *fetus* causes abortions in goats, sheep and cattle, and *C. hepaticus* causes spotty liver disease in layer hens [19,32]. The origin of these strains is of particular importance, and it has been proposed that *C. hepaticus* in chickens may have an environmental origin [33,34]. The widespread resistance of *Campylobacter* to β-lactams, tetracyclines, fluoroquinolones and macrolides emphasises the importance of ongoing susceptibility testing. The global crisis of multi-drug resistance (MDR) among bacteria is of significant public health concern, prompting efforts to restrict or eliminate antibiotic use across sectors, including their use in animal feed and for metaphylaxis. In 2019, the European Union implemented a regulation addressing the use of antibiotics in metaphylaxis [35]. Resistance to antibiotics can be acquired by activating a diverse set of molecular mechanisms, namely by inactivating the drug, altering the target of the antimicrobial, expressing antibiotic efflux pumps or decreasing membrane permeability [36].

**Figure 2:**
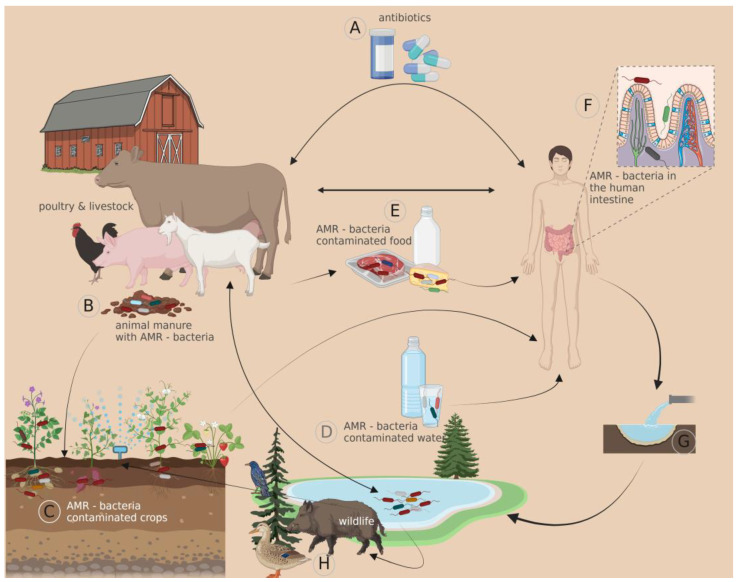
The role of livestock in the spread of antibiotic resistance in the environment. The administration of antibiotics to both human and animal populations favours the spread of resistance-associated genes in the natural environment, hence maintaining an ongoing reciprocal transmission, regardless of host origin. Created with Biorender.com and partly generated using Servier Medical Art and Twinkl.co.uk.

Antibiotic resistance in *Campylobacter* can be acquired through various mechanisms, including drug inactivation, target alteration, modification of the antibiotic efflux pumps and decreasing membrane permeability [36]. Macrolides resistance is mainly caused by point mutations of the 23S rRNA gene within its V domain or by the presence of the *cme*ABC operon encoding for multidrug efflux pump [37,38]. The presence of the *tet*O gene, which encodes a protective ribosomal protein, is generally associated with resistance to tetracycline [39]. Resistance to fluoroquinolones is frequently related to point mutation(s) in the DNA gyrase gene (*gyr*A) gene within the quinolone resistance-determining region (QRDR). Other genes associated with *Campylobacter* MDR are the *aad*E or *sat*4 (streptomycin/streptothricin resistance), *erm*B (erythromycin resistance), *aph*A-3 (aminoglycosides resistance) and *bla*_OXA-61_ (b-lactams resistance) genes [40]. Ultimately, these mechanisms contribute to antibiotic ineffectiveness and the development of multi-drug resistance. The overuse of antimicrobials in human medicine and the misuse of antibiotics in animal husbandry are significant contributing factors to the increased rates of antibiotic resistance among *Campylobacter* species [40]. All these antibiotic-resistance genes (ARGs) are also present in plants, animals, human hosts and generally in all natural environments, and their transmission and migration can be more dangerous than antibiotics (Figure 2).

The dissemination of these genes between host bacterial cells occurs through horizontal gene transfer (HGT) or vertical gene transfer (VGT). The HGT process relies on the transfer of genetic material via mobile genetic elements (MGEs) such as phages, plasmids, transposons and integrons [41,42]. MDR may be facilitated by these critical MGEs, particularly plasmids, which harbour drug-resistance genes and can be transmitted between bacterial populations [43]. The acquisition of plasmids can enhance the adaptive capabilities and ecological scopes of bacterial populations by providing new traits and modulating gene expression and mutation rates. The incorporation of plasmids is often accompanied by the acquisition of novel traits that allow bacteria, including pathogens, to inhabit specific environments [44]. Notably, unlike plasmid-mediated HGT, HGT mediated by phages is mainly limited within species because phage transmission is restricted by the similarity of the hosts’ genetic material [42,45].

The persistence of plasmids in bacterial populations has been studied due to their potential negative impact on host fitness. Empirical data suggests that plasmids can persist for extended periods, even in the absence of positive selection for their traits, which presents a challenge to understanding the mechanisms of plasmid persistence [46]. They also exhibit a wide range of sizes, with some being designated as “megaplasmids” due to their particularly large size. While there is no strict criterion for distinguishing megaplasmids from other large plasmids, the size threshold for what is considered a megaplasmid has varied [47].

Plasmid-encoded antibiotic resistance is a well-studied trait and is becoming increasingly important in clinical settings [47]. Consequently, the main mechanism for ARG spread is HGT. Antimicrobials have been broadly administered within farm animal husbandry and healthcare facilities to promote animal husbandry and prevent or treat bacterial infections. Consequently, this resulted in an overload of antimicrobials generated antibiotic residues in farm soils, sewage treatment plants, clinical settings and other sites [42]. The dissemination of ARGs through the environment via wastewater irrigation and manure application has the potential to impact plant and human health. Research has shown that antibiotic-resistant *E*. *coli* can migrate within plants and transfer ARGs through HGT. The use of antimicrobials in healthcare settings also leads to the occurrence of antibiotic-resistant bacteria in domestic wastewater, which can be a source of horizontal gene transfer of ARGs in municipal wastewater treatment facilities [41]. Moreover, it has been demonstrated that *E. coli* strains isolated from wastewater successfully transfer resistance genes and all together give rise to antibiotic resistance phenotypes [48].

The pathogenic character of *Campylobacter* resides in the diversity of its virulence factors, including invasion of epithelial cells, adhesion to the intestinal mucosa, toxicity, motility and chemotaxis, as well as surface structure [33,49]. Adhesion-related virulence factors include the *cad*F gene, encoding the *Campylobacter* adhesion protein to fibronectin (CadF), the *fla*A gene, encoding the major flagellin protein FlaA and the *doc*A gene, encoding a periplasmic cytochrome C peroxidase. Other adhesion-associated factors include the FlpA, PEB1, MOMP, HtrA, CapA and JlpA [33]. Among significant virulence markers linked with invasion encompass the *Campylobacter* invasion antigen conferred by the *cia*B gene, phospholipase A encoded by the *pld*A gene, invasion-associated marker (encrypted by the *iam* gene) and the *vir*B11 gene [40,50]. The flagella and chemotaxis factors are mainly involved in processes of mucosal colonisation, adhesion and invasion, as well as in biofilm formation. The surface structures associated with the virulence of *Campylobacter* spp. consist of capsular polysaccharide (CPS), lipooligosaccharide (LOS) and S-layer proteins. CPS participates in the invasion, adherence, intestinal colonisation and systemic infection, withstanding complement-mediated killing, while LOS is a mediator of invasion and adherence. LOS activates Toll-like receptor 4-mediated innate immunity and resists killing via cationic antimicrobial peptides. The S-layer proteins are relevant to immune evasion and resist complement-mediated killing [33]. Of all the toxins produced by *Campylobacter* spp., the most characterised is the cytolethal distending toxin (CDT) encoded by the *cdt*ABC operon. Some genes have been associated with GBS occurrence, namely *cgt*B and *wla*N genes, which both encode a β-1,3-galactosyltransferase enzyme involved in the production of sialylated lipooligosaccharide (LOS^SIAL^) [50].

## 3. Distribution of Virulence-Associated Genes across Different *Campylobacter* spp. Hosts

*Campylobacter* spp. was initially identified as a cause of animal disease in 1909, but it was not until 1980 that it was discovered that it also induces human infection [51]. This genus is commonly found in the intestinal tract of warm-blooded animals, including poultry, ruminants and swine, and can be transmitted to humans through the consumption of contaminated food or water or through close contact with infected animals [52]. Despite their inability to thrive outside the digestive tracts of homeotherms, pathogenic *Campylobacter* spp. is capable of surviving in food products for long periods of time. These bacteria are usually sensitive to environmental stress but have developed various mechanisms for survival in the environment and the food chain, which can lead to human infection [52]. Thermophilic *Campylobacter* spp. are widely distributed in the environment, which ultimately serves as an intermediate link between different hosts and habitats in the transmission of this bacterial pathogen [53,54]. Determining the virulence markers that contribute to the pathogenesis of campylobacteriosis is critical for identifying potentially more virulent strains and for gaining a deeper understanding of the mechanisms of infection [33,55]. Consequently, Table 1 shows a summary of genes related to virulence that have been detected in different *Campylobacter* spp. recovered from various animal and human hosts, including the environment.

## 4. Virulence and Antibiotic Resistance of *Campylobacter* spp. Poultry Isolates

Given the large spectrum of available data from poultry and because of the different approaches taken in these studies, connecting increased/decreased virulence with the levels of antibiotic resistance is difficult. *Campylobacter* spp. colonises the mucosa of the cecum and cloaca crypts in infected chickens and may also be present in other organs such as the spleen and liver [52]. A genomic analysis performed on *C. jejuni* isolates recovered from avian and human samples in Egypt revealed the presence of the *fla*A gene in all *C. jejuni* isolates, while the *cdt*A, *cdt*B and *cdt*C genes were present together in only ≈58.54% of the strains. None of the isolates displayed a concomitant presence of all the virulence genes screened (i.e., *fla*A, *cdt*A, *cdt*B and *cdt*C, *vir*B11, *wla*N, *iam*) in this study. Moreover, the *cdt*C gene revealed high similarity among *C. jejuni* isolates investigated, while the *iam* and *cdt*B genes showed the lowest similarity. Notably, the analysed strains unveiled 13 novel alleles. The *C. jejuni* strains obtained from the same host showed high heterogeneity, although the isolates recovered from chicken strains displayed the highest diversity [56]. Similarly, the virulence factors of 113 *C. jejuni* isolates obtained from poultry, and human sources were investigated by Ammar et al. (2021) in Egypt. All tested isolates unveiled the presence of the *fla*A gene, while 52% displayed the presence of the *vir*B11 gene, and 36% encoded the *wla*N gene [57].

Also, based on genome analysis of two fluoroquinolones (FQs) resistant *Campylobacter* chicken isolates (*C. jejuni* 200605 and *C. coli* 200606), it was revealed that strain 200605 is more virulent than strain 200606. This observation was based on the identification of *cad*F, *peb*A, *jlp*A (adhesion), *cia*BC (invasion), *cdt*ABC (toxin production), *flg*SR, as well as *ept*C (biofilm formation) genes only in the *C. jejuni* strain 200605. Similarities we only identified in regard to genes *che*AVW (chemotaxis), *gmh*, *waa*, *kps* (capsule formation), *kps*DEFCST (capsular genes) and *hld*E (LPS-associated gene). Notably, genes expressing capsular biosynthesis were detected in *C. coli* strain 200606, namely *Cj1416c*, *Cj1417c*, *Cj1419c* and *Cj1420c*, suggesting potential genetic exchange between *C. coli* and *C. jejuni* species [18]. The same authors analysed the presence of virulence factors in 55 *Campylobacter* (*C. jejuni* and *C. coli*) isolates originating from a layer farm in South Korea. The presence of the *cdt*B, *cst*II, *cad*F, *dna*J and *fla*A genes was reported in all of the isolates, while no strain was positive for the *ggt* gene. The prevalence of *csr*A, *pld*A and *cia*B genes varied between 33.3 and 95.9% [59]. All 55 tested isolates showed resistance to ciprofloxacin and nalidixic acid, while tetracycline resistance was observed in 93.9% and 83.3% of the *C. jejuni* and *C. coli* strains, respectively. Furthermore, all tested isolates showed susceptibility to sitafloxacin, as well as low erythromycin and gentamicin resistance and the presence of both *gyr*A gene point mutation and *tet*O gene was reported [59].

The antibiotic resistance and virulence factors of *C. jejuni* were also investigated in Brazilian slaughterhouse isolates from two different time periods (2011–2012; 2015–2016). The antimicrobial-resistant isolates, including those displaying multidrug resilience, were significantly more prevalent in 2011–2012, except for tetracycline. More prevalence rates were observed regarding *C. jejuni* isolation. However, the virulence genes revealed higher prevalence among the isolates from 2015 to 2016. All isolates were also tested for seven virulence-associated genes, namely *cia*B, *fla*A, *pld*A, *cad*F and *cdt*ABC genes. Approximately 83.6% of the 2011–2012 *C. jejuni* isolates harboured at least one of the screened virulence genes, whereas the 2015–2016 isolates revealed a significantly higher prevalence (97.7%). Despite the low prevalence rate of 2015–2016 *C. jejuni* isolates, these strains revealed increased virulence potential compared to the 2011–2012 group [62]. More recently, the same authors performed a comparative analysis of 44 *C. jejuni* isolates obtained from chicken carcasses and 20 *C. jejuni* clinical strains. Regardless of origin, 43.7% of all tested isolates harboured the *fla*A, *pld*A, *cad*F, *lux*S, *dna*J, *cbr*A, *cia*B, *cdt*ABC*, htr*A, *cst*II, *neu*A and *hcp* genes, while none of the analysed genomes displayed the presence of *pVir* genes. Their results indicated that the chicken isolates were more pathogenic than the human strains. The *cad*F, *pld*A and *cia*B genes showed higher prevalence among the isolates obtained from chicken carcasses. Moreover, the *ctd*ABC and *lux*S genes showed low prevalence among the clinical isolates, showing their presence in only 15% and 45% of human *C. jejuni* genomes [63].

The genetic elements of Gram-negative bacteria, such as integrons, may play an important role in the spread of resistance genes, given their presence in mobile genetic elements such as plasmids and transposons [93]. In South African *C. jejuni* chicken isolates, 26.92% of the tested isolates had more than two virulence-associated genes. For example, the *cia*B, *cdt*A*, cdt*B*, cdt*C, *pld*A and *cad*F genes revealed 23.1%, 26.9%, 30.8%, 42.3%, 34.6% and 23.1% prevalence among the *C. jejuni* isolates, respectively. Integrons, particularly class I and II, play a crucial role in the transmission of antibiotic resistance and are frequently associated with the Tn7 transposon family [94,95,96]. Class I integrons have been found in *Campylobacter*, but neither class II nor class III integrons have been detected in these species [97]. However, 92.3% of *C. jejuni* isolates displayed the presence of Class I integrons, whereas 65.4% of the tested isolates harboured Class II integrons [66].

An earlier comparative analysis regarding the aerotolerance, virulence-associated genes and antimicrobial resistance of *C. jejuni* isolates derived from chicken and duck meat showed that virulence factors were more prevalent in the *C. jejuni* strains obtained from retail chicken meat, while the duck meat isolates displayed a more increased prevalence of antibiotic resistance. Notably, the findings of this study were the first ones to present aerotolerant duck meat *C. jejuni* isolates. Particularly, the isolates obtained from duck meat displayed a more inferior prevalence for *cia*B and *cdt*B genes than chicken isolates, whereas the *virB11* gene showed low prevalence in both cases. The *iam* gene was harboured by 97.8% of the chicken isolates, while the *C. jejuni* isolates obtained from duck meat revealed its presence in 88.9% of the tested isolates. *Pld*A gene was present in 94.4% of the poultry isolates and 91.1% of the tested duck isolates. Both aerotolerant and hyper-aerotolerant *C. jejuni* strains isolated from duck meat revealed higher prevalence for the *cad*F, *peb*1, *pld*A and *doc*A genes in contrast to the oxygen-sensitive strains [74]. The prevalence of virulence markers in 220 *Campylobacter* isolates from ducks in southwestern China revealed that the most prevalent virulence-associated genes were *cad*F, *che*Y and *cdt*B, present in 100%, 92.7% and 92.3% of the isolates. Other highly prevalent virulence determinants consisted of *fla*A, *iam*A, *cdt*A, *cdt*C and *cia*B, harbouring 77.2%, 71.8%, 60%, 54.1% and 42.7% of the isolates of interest. The *vir*B11 gene was detected in only 7.7% of the isolates. Collectively, the *C. jejuni* strains displayed a higher number of virulence genes when compared to *C. coli* [75].

## 5. *Campylobacter* spp. Isolates Originating from Wild Birds

Wild birds can serve as vectors for the transmission of *Campylobacter* spp., either directly or indirectly, due to their high mobility. Many free-living and migratory birds serve as reservoirs for pathogenic *Campylobacter* species, which can cause illness in humans. While the risk of human infection from zoonotic agents in wild birds is low, it is considered a growing concern [80]. In a recent study, researchers collected 91 cloacal swabs from various wildlife waterfowl species to evaluate the genetic diversity and prevalence of *Campylobacter* spp. The *fla*A, *rac*R, *doc*A, *cad*F and *dna*J genes were present in all white-fronted geese and bean geese isolates, while a high prevalence (66.7–100%) was observed among both graylag geese and mallards. The *cia*B gene displayed high prevalence rates among bean geese, greylag geese, white-fronted geese and mallards isolates. Likewise, the *pld*A gene was also positively prevalent in the bean, white-fronted geese and mallards isolates. Only the strains originating from mallards sheltered the *vir*B11 and *iam* genes, showing 40% and 10% frequency, respectively. All isolates obtained from graylag, bean and white-fronted geese harboured the *cdt*B and *cdt*C genes, while only 55% and 75% of the mallard isolates carried these genes. The *Campylobacter* isolates originating from white-fronted geese, mallards, and greylag geese displayed the presence of the *cdt*A gene in a proportion of 80%, 60% and 33.3% of the investigated strains, respectively. The *cgt*B gene was carried by 20%, 33.3% and 20% of white-fronted geese, graylag geese isolates and mallards, respectively, while the *wla*N gene was only found in 5% of *Campylobacter* isolates originating from mallards. The results underlined that adhesion-associated genes (*fla*A, *cad*F, *rac*R, *dna*J and *doc*A) were highly prevalent among all tested isolates [50].

The variation of the *cdt*ABC genes among *Campylobacter* isolates originating from humans, wild birds and broiler chickens reveals a high diversity of the *cdt*ABC operon from the wild bird *C. jejuni* isolates, while both broiler and human isolates displayed high conservation of *cdt*ABC. The *cdt*A gene showed a higher number of nucleotide substitutions and was less prevalent among the tested *Campylobacter* isolates compared to both *cdt*B and *cdt*C genes. Moreover, non-functional *cdt*ABC operons were detected among the wild bird isolates. This study’s findings indicate that the *cdt*ABC operon plays a key role in host-specific colonisation efficiency [76]. Others analysed 60 *Campylobacter* isolates originating from 12 species of wild birds from South Korea. All wild bird-derived *Campylobacter* isolates showed a high occurrence of all 11 tested virulence genes, while no correlation between any particular sequence type and a specific virulence profile was observed. The *cad*F gene was present in 100% of the *Campylobacter* spp. isolates. All *C. coli* isolates harboured the *cdt*B and *cdt*C genes, while *C. jejuni* strains carried them in 98.1% and 94.3% of the tested isolates, respectively. The *wla*N gene was present in six *C. jejuni* isolates, while the *vir*B11 gene was carried by 11.3% of *C. jejuni* and 28.6% of *C. coli* strains [80].

A whole genome sequencing (WGS) analysis recently performed by Aksomaitiene et al. (2021) on seven (wild birds and cattle) *C. jejuni* isolates from Lithuania enabled the identification of virulent genes involved in adhesion (*cad*F and *peb*1), cytotoxicity (*cdt*A, *cdt*B and *cdt*C) and invasion (*yid*C and *yid*D). In addition to this, two *C. jejuni* strains unveiled the presence of nine phage-specific genes. Two of the isolates revealed the presence of *czc* (Zn resistance), *chr* (Cr resistance), *ncc* (Ni resistance) and *mer* (Hg resistance) genes. Also, both cationic antimicrobial peptide system and platinum drug resistance (*ctp*A) genes were found in three of the isolates. The genomes of three *C. jejuni* isolates revealed the presence of *vir*B2, *vir*B4, *vir*B8 and *vir*B9 genes responsible for the type IV-secretion system T4SS). *Trg*, *flg*E (chemotaxis and flagellar motility) and *bdl*A (biofilm dispersion) genes were also identified [81].

## 6. *Campylobacter* spp. Isolates Originated from Ruminants and Swine

*Campylobacter* spp. also colonises the intestines of cattle and can cause miscarriages in cows and sheep, although they are usually asymptomatic. *Campylobacter* spp. can also be found in lymphatic nodes, on the surface of hooves, and in the bristles of ruminants. Both meat and dairy products can be contaminated with *Campylobacter* spp., with raw milk being especially susceptible to infection [52,98]. There is a significant positive correlation between adherence and invasion of the isolates derived from cattle and swine. The low invasion level was correlated with the absence of the *vir*B11 gene. Moreover, the *iam* gene proved to be a trusted invasion-associated marker, and the gene pattern *vir*B11-*iam* was ascribed to the isolates that presented the highest invasion level. This study showed a high prevalence for adherence (*fla*A, *cad*F and *rac*R) and invasion (*vir*B11, *iam* and *pld*A) associated genes in *Campylobacter* spp. isolates from Poland [82].

The virulence and the prevalence of antimicrobial determinants were described for the first time in sheep on *Campylobacter* isolates obtained from Pernambuco, Brazil. Out of the 40 tested strains, 27.5% belonged to *C. jejuni*, 30% to *C. fetus* subsp. *fetus,* and 42.5% of them were *C. coli* strains [83]. The *cdt*A gene was found in 92.5% of all tested isolates, while *cdt*B and *cdt*C genes were harboured by ≈75% and 70% of the *Campylobacter* strains, respectively. The *cad*F and *rac*R genes also revealed high prevalence, namely 95% and 80%, while the *dna*J and *cia*B genes were present in only 47.5% and 20% of the isolates, respectively. The *pld*A gene was found in only one *C. jejuni* isolate, while none of the strains carried the *wla*N and *vir*B11 genes. Additionally, the C257T point mutation of the *gyr*A gene was present in all *Campylobacter* strains, while the A256G mutation and point mutations A2074G and A2075G of 23S rRNA were absent [83].

In a separate study performed on 98 *Campylobacter* spp. isolates from pork, beef, unpasteurised dairy products and raw milk, all isolates sheltered the *iam*, *rac*R, *cdt*B, *sod*B, *csr*A and *vir*B11 genes. The *wlaN* gene was present in all isolates, regardless of source origin, except *C. coli* isolates originating from pork meat. The highest prevalence rate was observed for the *cdt*B, *sod*B and *csr*A genes [84]. A similar investigation determined the prevalence rates and virulence genes of *Campylobacter* spp. identified an overall 83.3% prevalence in pigs and 16.1% in calves. The most frequent species detected were *C. jejuni*, except in pig samples, where *C. coli* was more prevalent. *C. jejuni* isolates carried the *cdt*ABC, *peb*3 (major antigenic peptide), *Cj0020c* (cytochrome C551 peroxidase) and *pgl*G (N-linked glycosylation gene) genes, while the *C. coli* isolates only harboured the *cdt*B and *cdt*C genes [79].

Interestingly, researchers from Portugal have identified a previously undescribed species of *Campylobacter* by analysing 5 isolates recovered from preputial bull mucosa originating from a beef herd with a reproductive failure medical history. The isolates displayed microaerophilic, catalase-negative, oxidase-positive, non-motile and Gram-negative characteristics. The identified bacteria were proposed as a novel *Campylobacter* species via *hsp60* and 16S rRNA genes analysis. The novel-defined *Campylobacter portucalensis* displays similarity to other *Campylobacter* species in terms of certain coding sequences, including genes encoding for adhesion and invasion, a type IV secretion system (T4SS) and antimicrobial resistance. The genome of *C. portucalensis* also revealed a high degree of homology with the T4SS of *C. fetus* subsp. *venerealis*. The pathogenic potential of *C. portucalensis* is currently unknown [99].

## 7. Recent Updates on Antibiotic Resistance among *Campylobacter* spp. Livestock Isolates

Recent data identified the presence of 18 distinct genetic determinants associated with antimicrobial resistance on either chromosomes or plasmids in *Campylobacter* spp. related to aminoglycosides, fluoroquinolones, β-lactams, lincosamides, phenicols, macrolides and tetracyclines. Compared to other *Campylobacter* species, *C. coli* genomes harboured both the highest number of antimicrobial resistance factors and a higher prevalence of multidrug genotypes. *C. coli* revealed the presence of 1 to 4 plasmids in 86.4% of the isolates, while *C. jejuni* isolates harboured 1 or 2 plasmids in only 14.1% of their genomes. Most antimicrobial resistance genes of *C. coli* were detected on plasmids, whereas other *Campylobacter* isolates revealed chromosomally encoded resistance. Collectively, only *C. jejuni*/*C. coli* isolates had antimicrobial resistance genes located on plasmids [100].

In poultry, fluoroquinolones and tetracycline resistance rates were higher among duck meat isolates compared to chicken isolates, while resistance to macrolides was detected in only a single duck *C. jejuni* isolate. Moreover, regardless of meat origin, gentamicin resistance was found in all tested isolates [74]. Earlier resistome analysis of 464 *Campylobacter* isolates obtained from chicken samples originating from various stages of the poultry production chain showed different patterns of virulence gene profiles between the *C. jejuni* and *C. coli* isolates. The *C. jejuni* isolates revealed the presence of 8 out of 9 investigated virulence factors, namely *cad*F, *cdt*ABC, *iam*A, *fla*A, *cia*B and *che*Y genes, while only three virulence-associated genes were detected in the *C. coli* isolates, including *vir*B11, *fla*A and *che*Y genes. Aminoglycoside resistance was highly prevalent among the tested isolates, revealing the presence of *aph*(3′)-III, *aph*(2″)-If, *ant*(6)-Ia, *aac*(6′)*-aph*(2″) and *aad*E-Cc genes. Genes encoding for β-lactamases resistance were diverse, including *bla*_OXA−456_, *bla*_OXA−184_, *bla*_OXA−460_ and *bla*_OXA−193_. All tested *Campylobacter* strains carried the *cat*A gene, while the *fex*A gene was reported in only two *C. coli* ST830 isolates. The *lnu*C gene was reported in 95% of the *C. jejuni* isolates, while the *emr*B gene was detected in only one *C. coli* ST872 isolate. The *tet*O gene was highly prevalent among both strains, while the *tet*L gene was present in 85% of the *C. jejuni* isolates and in only 15% of the *C. coli* isolates [61].

Likewise, the antimicrobial resistance determinants of 81 clinical *Campylobacter* strains from Santiago, Chile, revealed that C. *jejuni* and *C*. *coli* have 13 previously undescribed sequence types and alleles. All *Campylobacter* strains analysed showed the presence of the *cme*ABC operon, while a QRDR point mutation of the *gyr*A gene was present in only 53.1% of the isolates. The analysed strains maintained the *tet*O gene and a 23S rRNA mutation in 22.2% and 4.94% of the analysed strains. Notably, none of the strains unveiled the presence of the *erm*B gene, while 79% of the isolates displayed the presence of the *bla*_OXA-61_ gene [89]. Two clinical *C. fetus* isolates displayed resilience to streptomycin and sheltered aminoglycoside nucleotidyltransferase *ant*(6)-Ib. One of the isolates also revealed resistance to tetracycline and ciprofloxacin, conferred by the presence of the ribosomal protection protein *tet*44 and a mutation of the *gyrA* gene [101]. A recent WGS examination of 70 *Campylobacter* isolates presented a high genetic diversity among *C. jejuni,* and *C. coli* isolates derived from various ruminants (dairy cattle, beef cattle and sheep) from northern Spain. The *C. coli* isolates harboured various resistance encoding genes, such as *aph*(2″)-Ic, *aph*(3′)-III, *ant*(6)-Ia, *aad*E-Cc, *aad*9, *tet*O, *tet*O/M/O, *bla*_OXA-489_ and *bla*_OXA-193_ genes. The antimicrobial resistance genes detected in the *C. jejuni* isolates were *ant*(6)-Ia, *bla*_OXA-184_, *bla*_OXA-193_, *bla*_OXA-461_, *bla*_OXA-61_, *tet*O/32/O and *tet*O genes, as well as an *rps*L K43R point mutation in only one *C. jejuni* isolate. Both *C. jejuni* and *C. coli* isolates showed the presence of aminoglycoside resistance clusters; however, none maintained the *aad*E-Cc gene. Eight out of twelve *C. jejuni* plasmids showed high similarity with pTet plasmids, carrying the *tet*O and T4SS-associated genes. One *C. jejuni* isolate included a plasmid-encoded *tet*O/32/O gene, while eleven of them acquired tetracycline resistance via the plasmid-encoded *tet*O gene. Notably, evenly distributed pTet plasmids were observed among all tested *Campylobacter* isolates, regardless of host origin. Quinolone resistance was encrypted by *gyr*A gene point mutations, namely C257T and T86I, which conferred resistance to ciprofloxacin and nalidixic acid. The A2075G point mutation of the 23S rRNA gene provided erythromycin resistance to seven *C. coli* isolates, while none of the tested isolates displayed the presence of the *erm*B gene. Moreover, no *Campylobacter* isolate presented to have *cme*R and *cme*ABC operons [102].

In South African broilers, in 26 *C. jejuni* isolates, the highest antimicrobial resistance was detected to nalidixic acid (96.1%), tetracycline (80.7%) and erythromycin (84.6%), while resistance to ciprofloxacin was present in only 19.2%. The prevalence of the *cat*I, *cat*II, *cat*III, *cat*IV, *flo*R, *erm*B, *tet*O, *tet*A, *mcr-*4 and *amp*C genes were present in 84.2%, 78.9%*,* 52.6%, 10.5%, 52.6%, 73.7%, 68.4%, 47.4%, 42.1% and 10.5% of the tested isolates [66]. Similarly, antimicrobial resistance genes from 132 *Campylobacter (C. jejuni* and *C. coli***)** poultry isolates were favourably prevalent, ranging from 80 to 100% for the *cme*B, *tet*O and *bla*_OXA-61_ genes among all tested isolates, while the *aph*A-3 gene was not detected in any of the strains. The Thr-86-Ile mutation in the *gyr*A gene was detected in 80% of *C. coli* and 90% of *C. jejuni* strains. Moreover, the 23S rRNA A2075G point mutation was identified in 61% of *C. coli* and 86% of *C. jejuni* isolates, while the A2074C point mutation showed lower prevalence, holding by only 27% of *C. coli* and 14% of *C. jejuni* isolates. Notably, both A2074C and A2075G mutations were detected in 12% of the *C. coli* isolates [40].

## 8. Reports of Multi-Drug Resistant *Campylobacter* spp.

There have been numerous reports of multidrug resistance within the *Campylobacter* genus, regardless of the source of the isolates. Table 2 includes examples of antibiotic agents that have been identified in multidrug-resistant *C. jejuni*/*C. coli* strains isolated from both clinical and animal sources.

Previously investigated antimicrobial profiles and phylogenetic diversities of *C. coli* strains isolated from humans and swine between 2008 and 2014 in Osaka, Japan, identified that antimicrobial resistance (AMR) to erythromycin in the swine-derived strains was the highest. The swine strains that displayed erythromycin resistance harboured, besides a point mutation in the 23S rRNA, also the presence of the tetracycline resistance gene (*tet*O). The ST-1562 swine strain presented the highest frequency of multiple AMR, although this strain was not found in humans. Most of the human, poultry and cattle strains that displayed tetracycline resistance revealed the presence of the *tet*O gene on plasmids, yet swine strains displayed it on chromosomes [106]. The results of this study unveiled simultaneous antimicrobial resistance to at least three other antibiotic classes, namely quinolones, tetracyclines and β-lactams. Resistance to quinolones, macrolides and tetracyclines was observed in only one *C. coli* isolate [73].

The prevalence, virulence factors and antibiotic resistance profiles of 113 *C. jejuni* isolates obtained from Egypt’s poultry, and human sources also demonstrated a multidrug-resistant pattern, particularly to erythromycin and ampicillin. Approximately 96.5% of the isolates revealed resistance to five or more antibiotics [57]. Resistance to ciprofloxacin, cefixime and nalidixic acid was also identified in chicken isolates from Iran. Elevated antimicrobial resistance was identified to trimethoprim/sulfamethoxazole (80.8%), tetracycline (88.5%) and ampicillin (76.9%). Moreover, 80.2% of the tested isolates presented an increased multidrug resistance phenotype [58]. In a Chinese study, 220 *Campylobacter* isolates were classified as multidrug-resistant. *C. jejuni* showed relatively low resistance rates regarding gentamycin (15.6%), ciprofloxacin (13.8%) and nalidixic acid (25.7%), whereas the *C. coli* isolates displayed higher resistance rates to these antibiotics, namely 67.0%, 60.2% and 62.1%, respectively [75]. The genetic determinants associated with antimicrobial resistance were previously associated with point mutations into the *gyr*A gene or in the V domain of the 23S rRNA. Also, the presence of either the *bla*_OXA-61_ gene or the *cme*ABC efflux pump was also linked to the effect [90].

Phenotypic and genotypic characterisation of 541 *Campylobacter* isolates originating from various livestock at different stages of the production chain in North Carolina concluded that 90.4% of the tested isolates encoded antimicrobial resistance genes, while 43% revealed resistance to multiple antibiotics. Approximately 121 *gyr*A mutations were identified among poultry isolates, while only 9.7% belonged to the swine and cattle strains. All isolates showing high resistance to clindamycin, azithromycin and erythromycin also held the A2075G point mutation of 23S rRNA. Moreover, all *Campylobacter* isolates carrying *cme*ABC and *cme*R operons also carried the 50S L22 point mutation, while *C. coli* isolates had resistance genes related to aminoglycoside resistance [77].

Multi-drug resistance to antimicrobials was also identified in all tested *C. jejuni* isolates from broiler chickens in Brazil. The *cme*ABC operon and the *cme*G gene revealed high prevalence among the tested isolates, with 66.7% of the *C. jejuni* isolates showing the presence of *tet*O and *bla*_OXA-61_ genes, while only 30.43% of the strains carried the *aph*A-3 gene. All of the *C. jejuni* isolates harboured the Thr-86-Ile mutation in the *gyr*A gene and the A2075G point mutation in the 23S rRNA gene. Also, 85.7% of the *C. jejuni* strains included at least one plasmid [105]. Likewise, in broilers, in an Indian study, it has been identified that the highest antimicrobial resistance was detected to tetracycline, followed by macrolides, β-lactams, fluoroquinolones and sulphonamide, with a prevalence of 84.61%, 61.53%, 53.84%, 38.45% and 30.76% among all *Campylobacter* isolates. All the chicken isolates were resistant to tetracycline, while β-lactam, fluoroquinolone and macrolide resistance were detected in 66.66%, 25% and 33.33% of the tested isolates. Moreover, the isolates displaying resistance to macrolide and ampicillin carried the *bla*_OXA-61_ and 23SrRNA genes in 50% and 25% of the isolates [67]. Also, all 48 tested isolates from north-eastern Tunisia also displayed multi-drug resistance. Resistance to erythromycin, ciprofloxacin, gentamicin, chloramphenicol and nalidixic acid was detected in 97%, 60%, 30.3%, 72.7% and 87.9% of the *C. jejuni* isolates, respectively, while *C. coli* isolates displayed 100%, 86.7%, 20%, 80% and 80% resistance rates to the same antimicrobials. All screened antimicrobial resistance genes, namely *tet*O, *tet*A, *tet*L, *tet*B, *cme*B, *erm*B, *aph*A-3 and *bla*_OXA-61_ genes, were detected among the tested *Campylobacter* isolates. Moreover, the C257T point mutation in the *gyr*A gene and the A2075G point mutation in the 23SrRNA gene were identified [68].

A similar study investigated the antimicrobial resistance genes of *Campylobacter* isolated from broilers, calves, pigs and humans in Latvia. The *C. jejuni* isolates unveiled high resistance to nalidixic acid (94%) and ciprofloxacin (93.6%), while *C. coli* strains presented the highest resistance to streptomycin (73.5%). Low resistance to erythromycin and gentamicin was observed in *C. coli* and *C. jejuni* isolates. Circa 9.5% of the *C. jejuni* isolates obtained from calves displayed multi-drug resistance, while the human isolates showed a 16.7% prevalence of multi-drug resistance. Moreover, the *C. coli* isolates revealed multi-drug resistance in 29.6% of the pig isolates and in one human isolate and three calves isolates. All 30 quinolones-resistant *Campylobacter* strains possessed a *gyr*A gene point mutation, while all tetracycline-resistant bacteria showed the presence of the *tet*O gene. Aminoglycoside resistance was conferred by the *ant*(6)-Ib and *aph*(3′)-IIIa genes. The *cme*ABC operon was detected in all sequenced isolates. Notably, the *bla*_OXA-184_ gene was detected in five clinical isolates and one pig isolate. Further analyses linked these two strains by clustering them together [79]. A similar trend in observations was observed in a Belgian study which determined the antimicrobial resistance phenotypes and genetic diversity of 59 *C. coli* isolates. Some of the *C. coli* isolates that displayed erythromycin-resistance carried the *erm*B gene, point mutations in the *rpl*V and *rpl*D ribosomal genes, 23S rRNA A2074G point mutation and the *cme*ABC operon [109]. It is indeed obvious that the multidrug resistance in *C. jejuni* or *C. coli* isolates originating from poultry is most often associated with gene mutations or the presence of plasmids encoding for such mutations indicating that surveillance and close monitoring of AMR in such isolates is necessary.

## 9. Conclusions

It has been observed that various species within the *Campylobacter* genus can exchange genetic material with one another, as well as with other bacterial genera. This facilitates the transfer of genes that enhance the virulence of *Campylobacter* and allow it to withstand exposure to antibiotics. This bacterial genus is widely present in multiple hosts, including humans, animals, food products and the environment. The interconnected nature of these factors presents challenges for effectively addressing the potential negative consequences for human and animal health. Altogether the findings of this study emphasise the need for monitoring the relationship between virulence factors and antimicrobial resistance in *Campylobacter* spp. regardless of host origin.

## Figures and Tables

**Table 1:** Highly prevalent virulence-associated genes detected in *Campylobacter* spp. isolated from various sources.

Source	Virulence-Associated Genes	Gene Function	References
Poultry	* fla*A, *cad*F, *peb*A, *jlp*A, *rac*R, *doc*A, * dna*J	adhesion & colonisation	[18,19,40,56,57,58,59,60,61,62,63,64,65,66,67,68,69,70,71,72,73,74,75,76,77,78,79]
	* pld*A, *cia*BC, *iam*, * csr*A, *cbr*A, *ceu*E	invasion	
	*cdt*ABC	cytotoxicity	
	*flg*SR, *flg*E, *flg*H, *flg*L, *flh*A, * fli*A, *fli*F, *fli*M, *fli*Y, *mot*A, *pse*G, *Cj0371*, *Cj0358*, *Cj1371*, *che*Y, *che*AVW	motility & chemotaxis	
	*lux*S, *ept*C	biofilm formation	
	*htr*A, * per*R, *ca*tA	stress response	
	*neu*ABC, *gmh*, *waa*, *kps*, *kps*DEFCST, * Cj1420c*, *Cj1419c*, *Cj1417c*, *Cj1416c*, *kpsm*	CPS	
	*hld*E	LPS	
	*cst*II, *cst*III, *waa*C, *waa*F, *waa*V, *wla*N	LOS	
	*vir*B11	T4SS	
	*hcp*, *clp*B	T6SS	
Wild birds	*fla*A*, cad*F, *peb*1, *rac*R, *docA*, *dnaJ*	adhesion & colonisation	[50,60,76,80,81,82]
	*cia*B, *iam*, *yid*C, *yid*D	invasion	
	*cdt*ABC	cytotoxicity	
	*flh*A, *Trg*, *flg*E	motility & chemotaxis	
	*bdl*A	biofilm dispersion	
	*cgtB*, *wla*N	LOS	
	*vir*B11	T4SS	
Ruminants & Swine	*fla*A, *cad*F, *peb*1, *peb*3, *rac*R, *dnaJ*, *Cj0020c*, *pgl*G	adhesion & colonisation	[77,78,79,81,82,83,84,85]
	*iam*, *pldA*, *yid*C, *yid*D	invasion	
	*cdt*ABC	cytotoxicity	
	*trg*, *flg*E	motility & chemotaxis	
	*bdl*A	biofilm dispersion	
	*sod*B, *csr*A	stress response	
	*neu*ABC	CPS	
	*cst*II, *cst*III	LOS	
	*virB11*	T4SS	
Environment	*cad*F	adhesion	[85,86,87]
	*iam, cia*B	invasion	
	*cdt*ABC	cytotoxicity	
	*flg*R	motility	
	*pse*E, *lux*S	biofilm formation	
	*rrp*B	stress response	
Humans	*fla*A, *cad*F, *jlpA*, *pebA*, *rac*R, *dna*J, *aph*C, *pan*BCD	adhesion & colonisation	[55,56,57,63,65,69,72,79,87,88,89,90,91,92]
	*pld*A, *cbr*A, *cia*B, *iam*B, *iam*A	invasion	
	*cdt*ABC	cytotoxicity	
	*flg*E,* flhB*, *flgB*, *flaB*, *fla*C, *docC*	motility & chemotaxis	
	*htr*A	stress response	
	*neu*A, *neu*B1, *neu*C1	CPS	
	*Cj1136*, *Cj1137c*, *Cj1138*, *cst*II, *cst*III, *cgtB*, *wlaN*	LOS	
	*porA*	* MOMP *	
	*vir*B1, *vir*B11	T4SS	
	*clp*B, *hcp*	T6SS	

**Table 2:** Examples of antibiotic agents present in multidrug-resistant *C. jejuni*/*C. coli* recovered from clinical and animal origins.

Antibiotic Class	Antibiotic Agent (Resistance-Associated Genetic Determinants)	Origin	References
Aminoglycosides	gentamicin, kanamycin, spectinomycin, streptomycin (*aph*(2″)-Ii_1_, *aph*(2″)-Ii_2_, *apm*A, *aad*E*-sat*4*-aph*A-3, *aph*(3′)-III, *spw*, *aad*9, *aad*E)	Clinical	[20,90,103,104]
	spectinomycin, gentamicin, kanamycin, hygromycin, streptomycin (23S rRNA C1273T point mutation, *cme*ABC, *cme*G, *pmr*A, *hph*)	Animal	[68,75,81,103,104,105]
Macrolides	erythromycin, lincosamide (23S rRNA mutation, *lnu*G)	Clinical	[90,103,104]
	erythromycin, clindamycin, azithromycin, lincosamide (*lnu*C, 23S rRNA A2075G mutation, *erm*B, *cfr*C)	Animal	[34,57,67,68,75,77,81,104,106,107]
Tetracyclines	tetracycline (*tet*O, pTet-like plasmid)	Clinical	[20,57,79,90,106,108,109]
	tetracycline, doxycycline (*tet*O, *tet*M, *tet*A, *tet*L, *tet*B)	Animal	[18,32,57,67,73,75,79,81,104,105,107,109]
Quinolones	ciprofloxacin, nalidixic acid (*gyr*A gene T86I point mutation)	Clinical	[20,57,79,90,108,109]
	ciprofloxacin, nalidixic acid (*gyr*A gene C257T point mutation, *cme*ABC*, cme*G)	Animal	[18,32,57,58,67,68,73,75,79,81,104,105,107,109]
β-lactams	ampicillin (*bla*_OXA-193_)	Clinical	[57,90,108]
	ampicillin, ceftriaxone, cefixime, cefpodoxime, cefoxitin, oxacillin, cefepime (*bla*_OXA-448_, *bla*_OXA-61_, *bla*_OXA-133_, *ykk*C, *yaf*P)	Animal	[57,58,67,73,81,105]
